# Furfural Produces Dose-Dependent Attenuating Effects on Ethanol-Induced Toxicity in the Liver

**DOI:** 10.3389/fphar.2022.906933

**Published:** 2022-06-08

**Authors:** Zhuo Cheng, Xuanmei Luo, Zixin Zhu, Yonghui Huang, Xiue Yan

**Affiliations:** ^1^ The Department of Gastroenterology and Hepatology, Peking University Third Hospital, Beijing, China; ^2^ National Center of Gerontology, Beijing Hospital, Beijing, China; ^3^ Department of Infectious Diseases, The Affiliated Hospital of Guizhou Medical University, Guiyang, China

**Keywords:** furfural, NAD^+^, mitochondrial function, PI3K-Akt pathway, alcohol-associated liver disease

## Abstract

**Background:** Alcohol-associated liver disease (ALD) increases the health burden worldwide, but effective drugs to prevent ALD are lacking. Furfural is a small molecule that can limit alcohol production in microorganisms and may have the capacity to attenuate ethanol-induced toxicity.

**Methods:** Human HepG2 cells were incubated with ethanol and furfural, and cell viability, NAD^+^/NADH ratio, and mitochondrial function assays were performed. RNA sequencing (RNA-seq) data were used to annotate enriched pathways, and these findings were confirmed by reverse transcription-quantitative PCR (RT–qPCR) and Western blotting. C57BL/6J mice were fed a Lieber-DeCarli liquid diet. After 4 weeks, biochemical analysis of mouse serum and histological analysis of mouse livers were performed.

**Results:** Different concentrations of furfural exerted different effects on mitochondria: low-dose furfural reduced reactive oxygen species (ROS) production, maintained mitochondrial transmembrane potential, and inhibited apoptosis pathway activation, while high-dose furfural led to the opposite effects. In mice, furfural mitigated transaminase increases and attenuated the lipid metabolism disorder that had been induced by ethanol.

**Conclusion:** Low-dose furfural reduced ethanol-induced toxicity in the liver. Consuming food or beverages containing the appropriate level of furfural when drinking alcohol may be a convenient and useful way to prevent ALD.

## Introduction

Alcohol abuse increases the large current burden of health costs and expenditures committed to preventing social harm (Rehm et al., 2009). Alcohol use accounts for 2.2% and 6.8% of age-standardized deaths of females and males, respectively, worldwide (Griswold, 2018). Alcohol-associated liver disease (ALD) is related to alcohol intake and manifests as pathophysiological and histological changes in the liver. With increases in time and alcohol consumption, the liver undergoes several histopathologic changes ranging from steatosis to steatohepatitis, liver fibrosis, cirrhosis, and liver cancer (Fuster and Samet, 2018). Although abstinence can attenuate histological changes in alcoholic hepatitis (Parés et al., 1986), alcohol consumption is a severe problem in many regions, notably, in South Asia (Griswold, 2018; Im et al., 2020). A lack of drugs for treating ALD has led to an urgent search for an effective way to prevent this disease (Singal et al., 2018).

Alcoholic beverages may contain organic substances that can attenuate ethanol-induced toxicity. In addition, different types of alcoholic beverages may lead to differences in disease progression, for example, drinking beer or liquor is more likely to induce liver disease than drinking wine (Becker et al., 2002; Askgaard et al., 2015). In some studies, different types of liquor have been compared, and the results suggested that different types of liquors may affect the liver to varying degrees (Wu et al., 2002). Furthermore, some natural ingredients in consumables, such as coffee, may help reduce the hepatic damage caused by alcohol (Goh et al., 2014). Therefore, certain components in food or drinks may relieve the damage induced by ethanol consumption.

Furfural, an intermediate in the Maillard reaction, is a small molecule, that is, common in food and beverages, including alcohol. It is also an important byproduct that limits the ethanol generation rate in the fermentation of lignocellulosic hydrolyzates (Sárvári Horváth et al., 2003; Liu, 2006). The mechanism by which ethanol production is limited is mainly related to the redox state of cells. During metabolism, furfural consumes reduced nicotinamide adenine dinucleotide (NADH) and thus increases the nicotinamide adenine dinucleotide (NAD^+^)/NADH (Wahlbom and Hahn-Hägerdal, 2002) ratio. Overexpressing NADH-dependent oxidoreductase facilitates an increase in furfural tolerance in *E. coli*, indicating that furfural may rebalance the NAD^+^ redox state (Wang et al., 2011). A study with *Saccharomyces cerevisiae* showed that many genes related to mitochondria were changed after furfural treatment (Li and Yuan, 2010). Moreover, studies on the microbiome suggested that furfural metabolism may affect the redox state of cells (Wierckx et al., 2011; Ask et al., 2013). Although furfural is contained in most alcoholic beverages (Alcázar et al., 2006), few studies have focused on the relationship between furfural content in beverages and the effect of ethanol on mammals.

During ethanol metabolism, alcohol dehydrogenase (ADH) and aldehyde dehydrogenase (ALDH) degrade ethanol in the cytosol and acetaldehyde in the mitochondria, respectively (Cederbaum, 2012). These two processes require NAD^+^ as a coenzyme, and NAD^+^ is transformed to NADH during alcohol oxidation (Cederbaum, 2012). The reduction of NAD^+^ levels may limit glycolysis, fatty acid β-oxidation, and tricarboxylic acid cycle and exacerbate aging-related diseases, as does the high concentration of NADH. Meanwhile, mitochondrial NADH availability influences NADPH production by the nicotinamide nucleotide transhydrogenase and thus influences glutathione metabolism and response to oxidative stress (Katsyuba et al., 2020). Altering the NAD^+^/NADH ratio can change the level of many NAD^+^-dependent enzymes, such as Sirtuin 3 (SIRT3) (Hirschey et al., 2010) and PPARα (Yue et al., 2021), influencing liver metabolism. Furthermore, the level of NAD^+^ and the number of mitochondria increase after alcohol intake, important adaptations of the body to enhance alcohol metabolism (Han et al., 2012). Hence, normalization of the NAD^+^ redox balance can stabilize mitochondria, which may be a key process in the alleviation of ALD.

Therefore, we hypothesized that, because ethanol degradation decreases the NAD^+^ level and influences the NAD^+^/NADH redox state, consumption of an appropriate amount of furfural can stabilize mitochondria by restoring the intracellular NAD+/NADH ratio, attenuating ethanol-induced toxicity in cells. In this study, we offer a new perspective on alcohol intake in daily life and insights into a new method of ALD prevention.

## Materials and Methods

### Animal Experiments

Female C57BL/6J mice (eight to 10 weeks old, six mice in each group) were purchased from Beijing Vital River Experimental Animal Co., Beijing, China, and treated with Gao-binge alcohol. First, all mice were acclimated to the Lieber-DeCarli liquid control diet (710,260, Dyets) for 1 week. Then, the ethanol group (EtOH) mice were fed a Lieber-DeCarli ethanol liquid diet (in terms of energy, 36% is derived from ethanol, 11% is derived from carbohydrates, 17% is derived from protein, and 36% is derived from fat) for an additional 4 weeks. Ethanol with a low concentration of furfural (20 μmol/L) was given to the EtOH + LF group mice, and ethanol with a high concentration of furfural (640 μmol/L) was given to the EtOH + HF group mice. The EtOH + LF and EtOH + HF mice were subjected to these treatments for 4 weeks after the acclimation period. The high concentration of furfural group (HF) mice were fed a Lieber-DeCarli control liquid diet with a high concentration of furfural (640 μmol/L). The control (CTRL) mice were paired to each group and fed a Lieber-DeCarli control liquid diet. Mouse body weight was measured weekly. Twelve hours after the last feeding, the mice were anesthetized with pentobarbital sodium, and blood samples were collected from the orbital sinus. The mice were sacrificed by cervical dislocation. Isolated liver samples were immediately frozen or fixed in formalin for subsequent analyses. All animals received humane care. All procedures were approved by the Peking University Biomedical Ethics Committee Office (LA2022009).

### Cell Culture and Treatment

The HepG2 human hepatoma cell line (ATCC, United States) was cultured in Dulbecco’s modified Eagle’s medium (DMEM, HyClone, United States) supplemented with 10% fetal bovine serum (FBS, HyClone, United States) in a humidified incubator containing 5% carbon dioxide at 37°C. The HepG2 cells were seeded into 96-well microplates or 6-well microplates and incubated overnight. Then, the culture medium was replaced with fresh DMEM, and the cell cultures were allocated into four different groups: a control group [CTRL, incubated with phosphate-buffered saline (PBS)], an ethanol group (EtOH, incubated with 400 mmol/L ethanol), an ethanol with low furfural supplementation group (EtOH + LF, incubated with 400 mmol/L ethanol and 10 mol/L furfural), and an ethanol with high furfural supplementation group (EtOH + HF, incubated with 400 mmol/L ethanol and 320 μmol/L furfural); each culture was treated as indicated for 4 days.

### Cell Viability Assays

HepG2 cells were seeded into 96-well microplates at a density of 5 × 10^3^ cells per well, and these cultures were separated into four different groups as previously described. After treatment, 10 μl of Cell Counting Kit-8 (CCK-8) solution (Meilunbio, China) was added to each well, and the plates were incubated for 60 min at 37°C in the dark. The absorption of each well was measured at a wavelength of 450 nm with a configurable multimode microplate reader (Synergy H1, Bio-Tek, United States).

### NAD^+^ and NADH Assays

HepG2 cells were seeded into 6-well microplates at a density of 5 × 10^4^ cells per well. After treatment, the cells in each well were lysed with 200 μl of lysis buffer according to instructions of NAD^+^/NADH assay kit (Beyotime, China, S0175). Then, 20 μl of the suspension containing the lysed cells was added to a 96-well plate for detecting total absorption values of NADH and NAD^+^. For NADH detection, the lysed cell suspension was incubated at 60°C for 30 min, and then, a 20 μl aliquot of the suspension was added to the wells of a 96-well plate. Then, 90 μl of an alcohol dehydrogenase working solution was added to the plate wells and incubated at 37°C for 10 min. Then, 10 μl of a chromogenic solution was added and incubated at 37°C for 30 min. The absorption values of the wells were measured at a wavelength of 450 nm with a configurable multimode microplate reader (Synergy H1, Bio-Tek, United States). The values of NAD^+^ is obtained by calculating the difference between the total values and the values of NADH in the same sample.

### Detection of Reactive Oxygen Species Generation

Intracellular ROS detection was examined using 5-(and-6)-carboxy-2′, 7′-dichlorodihydrofluorescein–diacetate (carboxy-H2DCF–DA, Thermo Fisher Scientific, United States). After treatment, HepG2 cells were loaded with 10 μM carboxy-H2DCF–DA in warm Hank’s buffered salt solution (HBSS, Thermo Fisher Scientific, United States) for 30 min and washed twice with PBS (Meilunbio, China). Subsequently, the cells were viewed with a confocal fluorescence microscope Eclipse Ti inverted microscope (NIKON, Japan) at an excitation wavelength of 488 nm and an emission wavelength of 525 nm and analyzed with a configurable multimode microplate reader (Synergy H1, Bio-Tek, United States).

### Mitochondrial Membrane Potential Assay

For early apoptosis detection, a JC-1 mitochondrial membrane potential (ΔΨm) assay kit (Abcam, United Kingdom) was used to analyze the ΔΨm in each of the four cell treatment groups. After treatment, HepG2 cells were washed once with x1 dilution buffer and stained with 20 μM JC-1 dye in x1 dilution buffer at 37°C for 10 min in the dark. Excess JC-1 dye was then removed. The cells were washed twice with x1 dilution buffer, and 100 μl of DMEM was added to the previously treated wells to enhance cell recovery. The red fluorescence at excitation (535 nm)/emission (590 nm) wavelengths and green fluorescence at excitation (475 nm)/emission (530 nm) wavelengths were measured in the treated and dyed cells with a configurable multimode microplate reader (Synergy H1, Bio-Tek, United States). The background fluorescence was subtracted from the fluorescence of the treated cells, and then, the ratio of red fluorescence (indicating a polarized membrane) to green fluorescence (indicating a depolarized membrane) was obtained. In addition, we analyzed the fluorescence with a confocal fluorescence microscope (Eclipse Ti inverted microscope, Nikon, Japan).

### Western Blot Analysis

Following the treatment described previously, proteins in the HepG2 cells were isolated through treatment with RIPA buffer (Meilunbio, China) with phosphatase inhibitor cocktail I (Meilunbio, China) on ice. The proteins were separated by SDS–PAGE and transferred to nitrocellulose membranes (Millipore, Billerica, United States). After blocking, the membranes were incubated overnight with antibodies at 4°C and then with horseradish peroxidase (HRP)-conjugated anti-rabbit and anti-mouse secondary antibodies. Antibodies against PI3K (ab191606), phosphorylated (p)-PI3K (ab182651), Akt (ab179463) and p-Akt (ab192623) antibodies were purchased from Abcam; antibodies against mTOR (66888-1-Ig), p-mTOR (67778-1-Ig), Bcl-2 (12789-1-AP), Caspase-9 (10380-1-AP), Bax (50599-2-Ig), and β-actin (66009-1-Ig), and the secondary antibodies for mouse (SA00001-1) and rabbit (SA00001-2) were purchased from Proteintech. Finally, the blots were visualized with a ChampChemi detection system (Sage Creation, China).

### RNA Isolation and RNA-Seq

Total RNA from HepG2 cells in the four different treatment groups was isolated with TRIzol™ reagent (Thermo Fisher Scientific, United States). A Dynabeads™ mRNA purification kit (Invitrogen) was used to extract mRNA from total RNA on the basis of the manufacturer’s instructions. The library was quantified with a Qubit dsDNA HS assay kit (Thermo Fisher Scientific, United States) and a Qubit 3.0 fluorometer (Thermo Fisher Scientific, United States) in preparation for a quality analysis before sequencing. The library was prepared according to the Illumina sample preparation guide. The DNA libraries were sequenced using the NextSeq platform (Illumina, United States) with 150-bp paired-end reads.

### RNA-Seq Read Mapping and Differentially Expressed Gene Analysis

The quality of the reads was assessed with FASTQC v0.11.8 software, and the Trimmomatic v0.38 tool was used to remove the low-quality reads and adaptors. Then, clean reads were mapped to a University of California–Santa Cruz (UCSC) reference genome (hg19) using the STAR v2.7.0 aligner. The aligned read files were processed by StringTie v1.3.3b on the basis of normalized RNA-seq fragment counts to indicate the relative abundances of genes in the transcriptome, which is reported herein as transcripts per million (TPM). Differential gene expression was assessed by the edgeR v3.32.1 package in R software, and the DEGs were chosen on the basis of FDR <0.05 and fold change > 1. A Principal component analysis (PCA) was carried out using the FactoMineR package to explore the transcriptome profiles between groups. The clustering was performed with the gplots (version 3.1.1) package, and the results are presented in a heatmap.

### Gene Ontology and Kyoto Encylopedia of Genes and Genomes Pathway Enrichment Analyses of the Differentially Expressed Genes

To analyze the GO and KEGG pathway information, the DEG lists were uploaded to the DAVID online tool for a series of analyses, and in these cases, *p* < 0.05 was considered to indicate significance.

### Validation of Results With Reverse Transcription–Quantitative PCR

To validate the different expression levels of the mRNAs of interest, specific primers were designed targeting the selected mRNAs; the sequences are shown in Supplementary Table S1. Total RNA from the HepG2 cells in the four different treatment groups was isolated with TRIzol™ reagent (Thermo Fisher Scientific). For RT–qPCR, cDNAs were prepared with M-MLV reverse transcriptase (Thermo Fisher Scientific, United States). The resulting cDNAs were subjected to real-time PCR analysis with gene-specific primers and a SYBR Green SuperMix kit (TaKaRa, Japan) on an MA-6000 real-time PCR detection system (Molarray, China). Gene expression was normalized to that of the GAPDH gene, and the relative expression levels of the mRNAs of interest were determined by the comparative threshold cycle (Ct) method using formula 2^−(ΔΔCt)^.

### Biochemical Analysis of Mouse Serum

After anesthetization, blood samples were collected from the retro-orbital sinus of the mice. The serum was centrifuged at 3000 rpm for 5 min. The serum biochemical parameters, including alanine transaminase (ALT), aspartate transaminase (AST), triglyceride (TG), total cholesterol (TC), low-density lipoprotein cholesterol (LDL-C), and albumin (ALB) levels, were analyzed on a Mindray BS-2000M automatic analyzer according to the manufacturer’s instructions in the detection kit purchased from Mindray Biomedical (Shenzhen, China).

### Histological Analysis of Mouse Livers

Oil red O staining was performed on liver cryosection slices. Images were captured with a light microscope (Leica, Solms, Germany) and a HITACHI HT7700 transmission electron microscope (HITACHI, Tokyo, Japan), respectively.

### Statistical Analysis

All experiments were performed with biological duplicates. Comparisons between groups in HepG2 cells or in mice were respectively performed by one-way analysis of variance (ANOVA) followed by least significant difference (LSD) test or two-way ANOVA followed by Bonferroni test. The data are reported as the means ± SEM. Differences were considered significant when *p* < 0.05. The experimental parameters were analyzed with SPSS software 26.0.

## Results

### Low-Dose Furfural Attenuated Ethanol-Induced Toxicity and Elevated the NAD^+^/NADH Ratio

**FIGURE 1:**
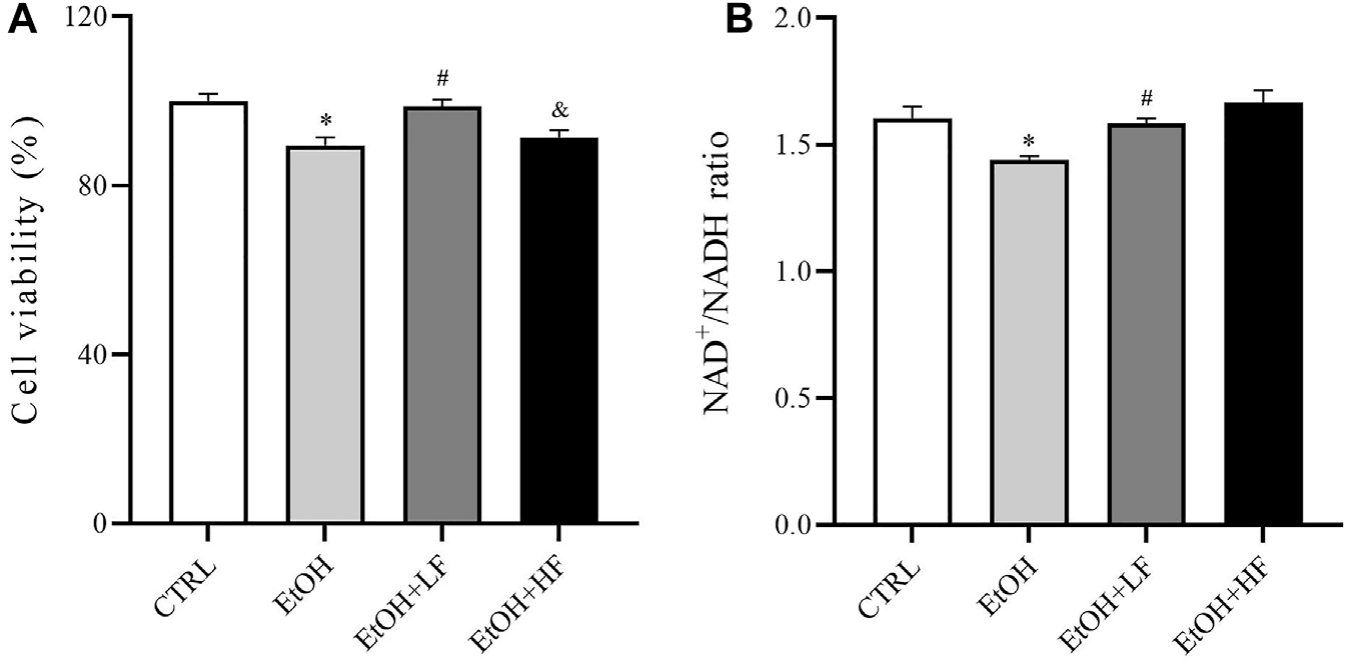
Furfural reversed changes induced by ethanol in HepG2 cells treated with ethanol for 4 days. **(A)** Cell viability test with cell counting kit-8 (CCK-8). **(B)** The ratio of NAD^+^ to NADH. *p < 0.05, compared with the CTRL group; ^#^ *p* < 0.05, compared with the EtOH group; ^&^ *p* < 0.05, compared with the EtOH + LF group. Abbreviations: NAD, nicotinamide adenine dinucleotide.

To determine whether furfural contributed to the reduction in ethanol-induced toxicity, we first determined cell viability after treatment with ethanol and furfural for 4 days. Surprisingly, the analysis revealed that compared with that in the CTRL group, the cell viability was significantly decreased in the EtOH group and EtOH + HF group, while that of the EtOH + LF was higher than that of the EtOH group (Figure 1A).

Then, we measured the ratio of NAD^+^/NADH in different groups (Figure 1B) to test the hypothesis that both furfural and ethanol influence the NAD^+^/NADH ratio in cells. As expected, the NAD^+^/NADH ratio in the EtOH group was significantly lower than that in the CTRL group, which was significantly reestablished by low-dose furfural treatment. There was no significant difference when the furfural dose was increased, but the NAD^+^/NADH ratio showed an increasing trend. These results indicated that the concentration of furfural may affect furfural effects.

### Low-Dose Furfural Attenuated Ethanol-Induced Mitochondrial Dysfunction

**FIGURE 2:**
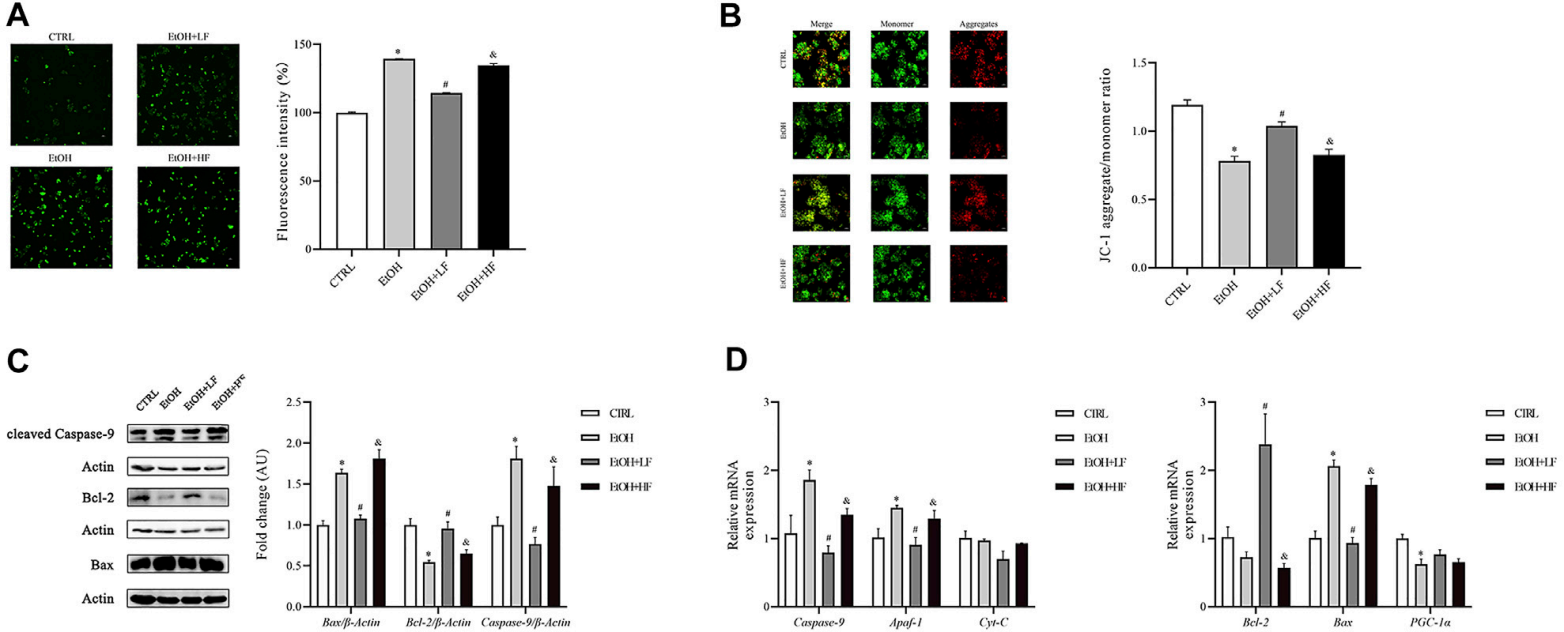
Furfural normalized mitochondrial function in HepG2 cells treated with ethanol. **(A)** Reactive oxygen species (ROS) levels as detected by DCFH-DA assay. The bar graph shows the fluorescence intensity of DCFH-DA representing the general ROS levels. **(B)** JC-1 assay. The bar graph shows the ratio of aggregated JC-1 (red)/monomeric JC-1 (green), representing the mitochondrial membrane potential (Δψm). Scale bar: 50 μm. **(C)** The relative expression of factors in the mitochondrial apoptosis pathway at the protein level. **(D)** The mRNA levels of factors in the mitochondrial apoptosis pathway and involved in oxidative stress damage, as measured by RT–qPCR. *p < 0.05, compared with the CTRL group; ^#^ *p* < 0.05, compared with the EtOH group; ^&^ *p* < 0.05, compared with the EtOH + LF group. Abbreviations: Apaf-1, apoptotic peptidase activating factor-1; CytC, cytochrome c.

Because the NAD^+^/NADH redox state may play an important role in cellular responses to furfural, we focused on the key sites of aerobic respiration: mitochondria. An immunofluorescence assay of ROS demonstrated that low-dose furfural reduced the ROS production induced by ethanol. Moreover, a high dose of furfural increased ROS production, which reached levels similar to those in the EtOH group (Figure 2A). Next, we focused on the ΔΨm. The analysis revealed that the ΔΨm was decreased in the EtOH and EtOH + HF groups compared to the CTRL group, and that value of the EtOH + LF was significantly higher than that of the EtOH group, suggesting that a low dose of furfural attenuated damage to the electron transfer chain (Figure 2B).

Considering that the decrease in mitochondrial transmembrane potential has been associated with mitochondria-mediated apoptosis, we detected the expression levels of Bax, Bcl-2, and caspase-9, which are important in apoptosis, by Western blotting (Figure 2C). In the EtOH and EtOH + HF groups, Bax and caspase-9 expression was upregulated, and that of Bcl-2 was downregulated. In contrast, in the EtOH + LF group, Bax, Bcl-2, and caspase-9 expression was similar to the control after normalization, and similar results were found for Bax, Bcl-2, and caspase-9 expression in each group by RT-qPCR (Figure 2D).

### Comparison of Gene Expression in Cells Treated With Different Furfural Concentrations

**FIGURE 3:**
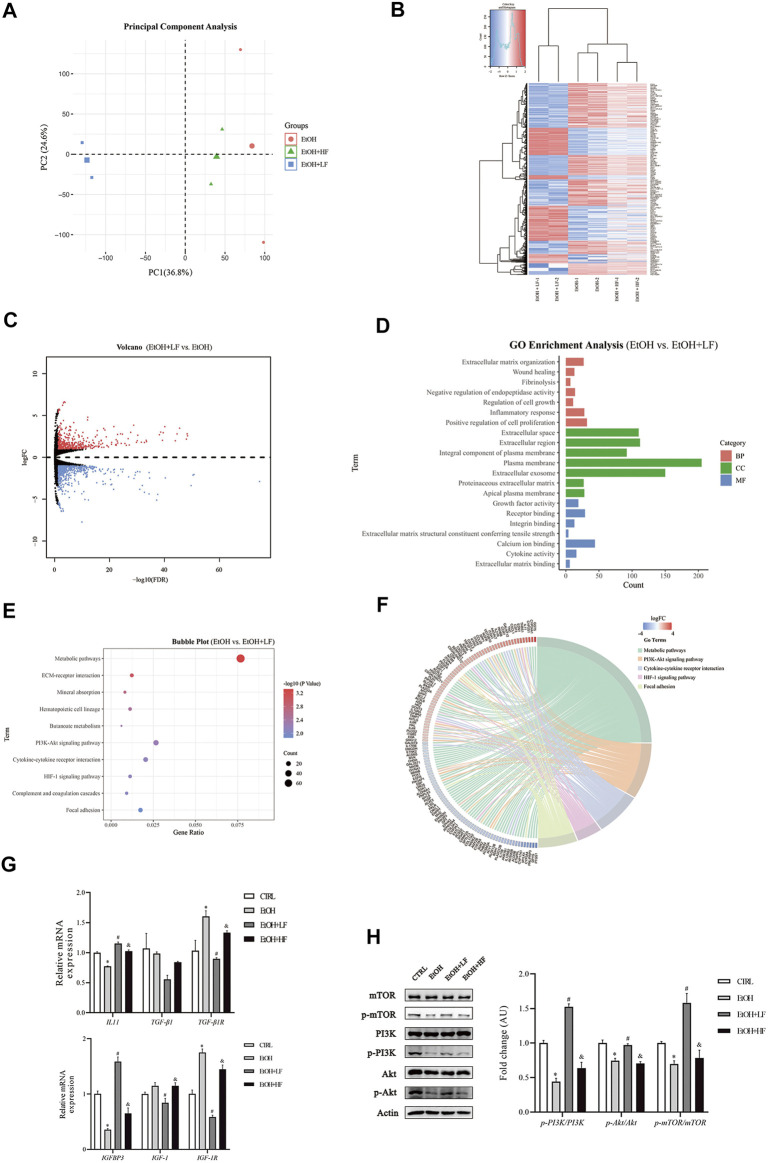
mRNA changes after furfural treatment of HepG2 cells. **(A)** Principal component analysis (PCA) score plot for three treated groups. **(B)** Heatmap showing the mRNA expression profiles between groups. Red stripes represent highly expressed genes, while blue stripes represent genes expressed at low levels. **(C)** Volcano map of DEGs between the EtOH group and EtOH + LF group. Red dots represent highly expressed genes in the EtOH + LF group, while blue dots represent genes expressed at low levels. **(D)** GO enrichment histogram between the EtOH group and the EtOH + LF group. **(E,F)** The KEGG pathway enrichment analysis between the EtOH group and EtOH + LF group. Chord diagram showing the annotated genes and their expression in enriched pathways. Red bars represent high gene expression in the EtOH + LF group, while blue bars represent low gene expression. **(G)** The mRNA levels of fibrosis-related factors as measured by RT-qPCR. **(H)** Phosphorylation levels of key molecules in the PI3K-Akt signaling pathway. The data are expressed as the mean ± SEM on the basis of three or four independent experiments. *p < 0.05, compared with the CTRL group; ^#^ *p* < 0.05, compared with the EtOH group; ^&^ *p* < 0.05, compared with the EtOH + LF group. Abbreviations: GO, Gene Ontology; KEGG, Kyoto Gene and Genomic Encyclopedia; BP, biological process; CC, cellular component; MF, molecular function. IGFBP3, insulin-like growth factor binding protein 3; IGF-1(R), insulin-like growth factor 1 (receptor); IL-11, interleukin-11; TGF-β1, transforming growth factor-beta 1; PI3K, phosphoinositide 3-kinase; mTOR, mammalian target of rapamycin.

To further elucidate the mechanistic role of furfural, we used high-throughput RNA-seq to identify different pathways in the three EtOH treatment groups. A total of 1,055 DEGs were identified between the EtOH + LF group and EtOH group (Supplementary Table S2), and 593 DEGs were identified between the EtOH + LF group and EtOH + HF group (Supplementary Table S3). These data were uploaded to DAVID online software, and the enriched GO categories and KEGG pathways were obtained. The results indicated that the EtOH and EtOH + HF groups showed similar gene expression patterns and that the DEG expression levels in the EtOH + LF group showed patterns opposite those of the other two groups (Figures 3A–C). A GO analysis of biological processes (BPs) enriched with the DEGs between the EtOH and EtOH + LF groups revealed positive regulation of cell proliferation, inflammatory response, and extracellular matrix organization. The GO cellular components (CCs) enriched with DEGs of these two groups were plasma membrane and extracellular. In the GO molecular function (MF) category, calcium ion binding, and protein homodimerization activity were enriched with these DEGs (Figure 3D). A KEGG pathway enrichment analysis revealed significant pathways including metabolic pathways, the PI3K-Akt signaling pathway, and cytokine–cytokine receptor interactions (Figure 3E). Compared with the EtOH group, in the EtOH + LF group, the expression levels of some genes which related to oxidoreductase activity, such as *CYP3A4*, *CYP3A5*, *BDH1*, *MAOB*, *CHDH*, and *DMGDH* were downregulated, and the expression levels of some acyl-CoA synthetase genes, such as *ACSM2B*, *ACSM2A*, *ACSM5* were downregulated, while *ACSL5* and *ACSL6* were upregulated (Figure 3F).

We performed RT–qPCR and Western blot analyses to verify the alterations revealed in the RNA-seq data. The expression of fibrosis-related gene TGF-β1R was significantly lower in the EtOH + LF group than in the EtOH group (Figure 3G). Interestingly, compared to the CTRL group, the level of IL-11 was downregulated in the EtOH group. A low concentration of furfural seemed to decrease the inhibition of IL-11 gene expression by ethanol. Compared with that in the EtOH group, the expression of IGF-1 and IGF-1R was significantly downregulated in the EtOH + LF group. IGFBP3, which can regulate IGF-1 function (Annunziata et al., 2011), showed a trend opposite that of IGF-1 and IGF-1R. Subsequently, we analyzed the PI3K-Akt-mTOR pathway and found that the phosphorylation levels of PI3K, Akt, and mTOR in the EtOH + LF group were higher than those in the EtOH group or EtOH + HF group (Figure 3H). We also detected the gene expression level of antioxidant enzymes. In the EtOH + LF group, the expression levels of SOD2 and CAT were significantly upregulated compared to the EtOH group (Supplementary Figure S4).

### Furfural Attenuated the Liver Damage Induced by Ethanol *In Vivo*

**FIGURE 4:**
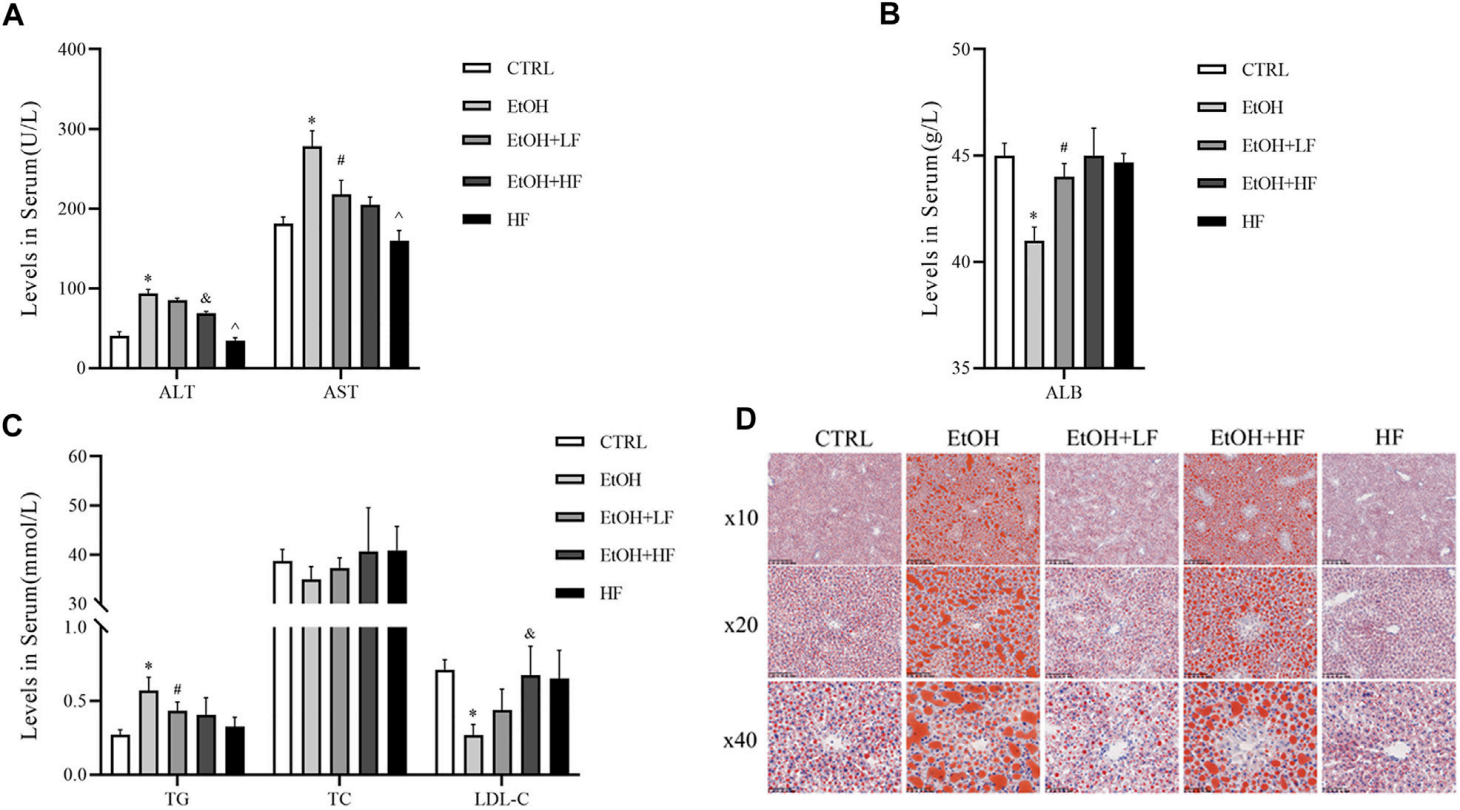
Furfural alleviated ethanol-induced liver injury and lipid metabolism disorder in mice. **(A)** Serum alanine transaminase (ALT), aspartate transaminase (AST) levels. **(B)** Serum ALB levels. **(C)** Serum lipid levels. **(D)** Oil red O staining of liver tissues. The data are expressed as the means ± SEM. *p < 0.05, compared with the CTRL group; ^#^ *p* < 0.05, compared with the EtOH group; ^&^ *p* < 0.05, compared with the EtOH + LF group; ^*p* < 0.05, compared with the EtOH + HF group.

Finally, we sought to confirm our speculation suggesting that a low furfural dose can detoxify ethanol in mice. There was significant interaction between ethanol and furfural for ALT, TG, LDL-C, and ALB. Corroborating our hypothesis, the ALT and AST levels were significantly elevated in the EtOH group compared with those in the CTRL group, and these levels were lower in the EtOH + LF group compared to those in the EtOH group (Figure 4A). To our surprise, the EtOH + HF group mice presented with lower transaminase levels, but the differences were not significant compared with that in the EtOH + LF group mice. The levels of ALT and AST in the HF group were lower than those in the EtOH + HF group. Compared to that in the CTRL group, the ALB level was significantly decreased in the EtOH group, and no significance in ALB level was observed in the other groups (Figure 4B).

As mitochondria are crucial for systemic lipid homeostasis (Benador et al., 2019), we assessed lipid metabolism in mice. The EtOH group showed a significantly lower level of LDL-C than the CTRL group. The downregulated expression was attenuated in the groups treated with furfural, and this effect was related to furfural concentration. Compared with that in the CTRL group, the TG level in the EtOH group increased dramatically, and this tendency was mitigated in the groups treated with furfural. The TC level was not notably different between groups (Figure 4C). Oil red O staining showed the accumulation of lipid droplets in the liver samples of the EtOH group, and low-dose furfural attenuated this change (Figure 4D). However, lipid droplet accumulation was increased in the EtOH + HF group. No significant lipid droplet accumulation was observed in the HF group.

## Discussion

ALD increases the health burden worldwide; therefore, finding ways to reduce ethanol-induced toxicity is important work. In this study, we demonstrated that low-dose furfural attenuated the toxicity induced by ethanol *in vitro* and *in vivo,* but increasing the furfural concentration did not enhance this effect. The potential mechanism of furfural may involve normalization of the NAD^+^/NADH ratio and mitochondrial function maintenance.

First, we found that furfural normalized cell viability that had been diminished by ethanol exposure, and this normalization function was significant at a low furfural concentration. The NAD^+^/NADH ratio increased with increasing furfural concentration, which was consistent with a previous study on bacteria (Tsuge et al., 2014; Gonzalez et al., 2018). Furfural has been widely studied in microorganisms as a major inhibitor of lignocellulose fermentation (Liu, 2006). Furfural treatment can significantly inhibit the production of ethanol (Lopes da Silva et al., 2017). Notably, a previous study on furfural metabolism in *Corynebacterium glutamicum* showed that furfural was oxidized to furoic acid or reduced to furfural alcohol in an aerobic state (Tsuge et al., 2014). With the increase in furfural concentration, furfural likely was reduced to furfural alcohol and was associated with higher proportions of NAD^+^ and NADP^+^ (Tsuge et al., 2014). A study with *Escherichia coli* showed that ethanol production can be promoted by regulated NADH-dependent oxidoreductase expression (Miller et al., 2009; Wang et al., 2011). Hence, the interaction between furfural and ethanol may be related to the NAD^+^/NADH redox state. From the results of RNA-seq analysis, some related pathways can be identified, such as steroid biosynthesis and steroid hormone biosynthesis in KEGG, oxidoreductase activity and monooxygenase activity in the molecular function GO annotation can be identified. These pathways may increase the consumption of NADH or NADPH, thus restoring the imbalance of the NAD^+^/NADH ratio induced by ethanol. Because the imbalance of this redox state is associated with mitochondrial dysfunction (Ying, 2008; Imai and Guarente, 2014), we speculate that the detoxification effect of furfural may be concentration-dependent and related to mitochondrial function.

To prove that low-dose furfural maintains the mitochondrial function, we measured ROS levels as a means to evaluate oxidative stress. The results suggested that low-dose furfural decreased intracellular ROS production that had been induced by ethanol, while a high furfural concentration did not reduce ROS levels. In addition, the expression levels of *SOD2* and *CAT* that could reduce ROS level (He et al., 2017) were also upregulated when treated with a low concentration of furfural. These results supported our hypothesis that the effect of furfural is concentration-dependent. Next, the ΔΨm assessment revealed a similar dependance on furfural concentration, confirming our hypothesis. Then, we measured the expression levels of Bax, Bcl-2, and caspase-9, which are biomarkers of mitochondrial-mediated apoptosis. We found decreased Bax and caspase-9 expression and increased Bcl-2 expression in the groups treated with a low-dose furfural, indicating that a low furfural concentration inhibited the mitochondrial apoptosis pathway. Therefore, an appropriate amount of furfural may enhance mitochondrial function and reduce the apoptosis rate.

Performing second-generation sequencing, we identified pathway and functional alterations and found that multiple metabolism-related pathways, such as the PI3K-Akt pathway and HIF-1 signaling pathways, were changed by furfural treatment. Then, we measured the phosphorylation levels of PI3K, Akt, and mTOR and thus confirmed the RNA-seq results. Low-dose furfural increased the ratio of these phosphorylated proteins to their unphosphorylated counterparts. According to previous studies, increasing expression of PI3K-Akt-mTOR pathway could block the apoptosis by elevating the expression of Bcl-2, as well as activate HIF-1α target genes (Majumder et al., 2004). Activation of the mTOR-HIF-1 pathway induced glycolysis and increased NAD^+^/NADH ratio (Cheng et al., 2014). Moreover, hypoxia and HIF-1 activation can downregulate CYP3A4 and CYP3A5 (Valencia-Cervantes et al., 2019). Furthermore. the regulation of acyl-CoA synthetase may be accomplished *via* the NAD^+^/NADH (Eaton, 2002). In general, ACSMs were more likely located in mitochondria while ACSLs were located in cytosol and mitochondria. And the subcellular location of these enzymes may play a role in the process that controlling lipid metabolism (Huh et al., 2020). Acyl-CoA synthetase activity could affect β-oxidation flux and partitioning of fatty acids between β-oxidation, triacylglycerol synthesis, and phospholipid synthesis. Low expression of ACSMs reduced the metabolism of medium-chain fatty acids, and high expressions of ACSLs could increase long-chain fatty acid oxidation and reduce the lipid droplets formation (Okada et al., 2016). We also found that many pathways related to the extracellular matrix were enriched with DEGs, indicating that furfural may affect the formation process of collagen, which has been previously identified as a key component in liver fibrosis (Schuppan et al., 2018). These findings indicated that different concentrations of furfural exert different effects and that among organelles, mitochondria may be the most affected by changes in furfural concentration (Cheng et al., 2010).

Finally, we found that furfural attenuated liver damage in ethanol-treated mice. Unexpectedly, in contrast to the cell experiment results, high-dose furfural tended to exert a greater influence on the reestablishment of normal transaminase and TC levels, which had been increased by ethanol exposure. The reason for these differences in cells and mice may be related to metabolic differences *in vitro* and *in vivo*. A high furfural concentration did not seem to exert a clear effect on the mouse livers, as determined through laboratory examinations. Another interesting finding is that LDL-C level in the ethanol group was lower than that in the normal group. The literature indicates similar changes (Taskinen et al., 1987; Brinton, 2012), and furfural may attenuate this alteration. Oil red O staining revealed that furfural inhibited lipid droplet formation, confirming that furfural can attenuate ethanol-induced lipid metabolism disorders. This outcome may be explained by rodents having a better tolerance than cells to furfural. Although, to the best of our knowledge, the metabolic outcomes were somewhat different from those observed in microorganisms, studies with humans and rodents showed that furfural was primarily oxidized to 2-furoic acid, which binds to acetyl-CoA and glycine, and was ultimately eliminated in urine as N-(2-furfuryl)-glycine (Parkash and Caldwell, 1994; Joint FAO/WHO Expert Committee on Food Additives, 2004). Considering the abovementioned results, we assume that furfural may be transformed into an active substance, and we will continue to explore the specific active metabolites of furfural metabolism in cells in the future.

Besides, 5-hydroxymethyl-2-furfural (5-HMF) is also a product of the Millard reaction as furfural but has less accumulation in the fermentation process (Barakat et al., 2012). The effects of 5-HMF were controversial (Han et al., 2017; Qiu et al., 2022). One possible reason may be that the concentrations of 5-HMF were varied in different studies. From our results, the concentration of these substances may play an important role in the ability to protect against poisons like ethanol.

**FIGURE 5:**
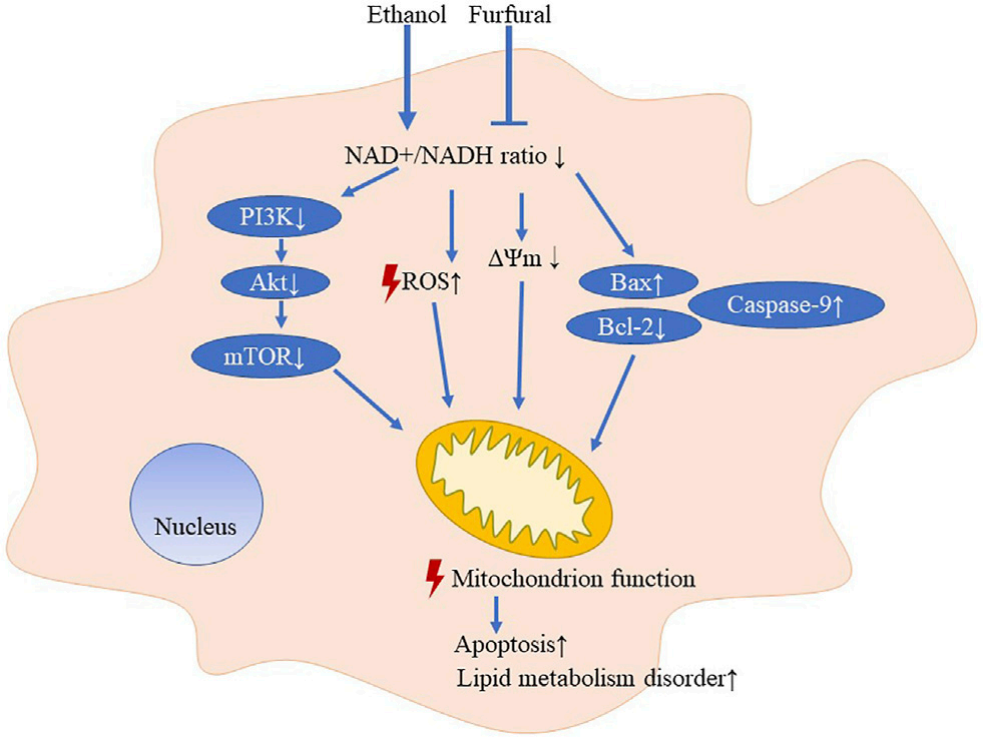
Pattern diagram of low-dose furfural in attenuating effects on ethanol-induced toxicity.

Furfural is a food additive with a unique scent and is found at relatively high concentrations in coffee (about 2–20 μmol/L) (Chaichi et al., 2015), hot chocolate (about 50 μmol/L) (Maldonado-Mateus et al., 2021), and other baked goods. According to our findings, appropriate intake of certain foods or beverages with a low furfural concentration in conjunction with drinking alcohol may help to improve the ethanol metabolism in the body and thus prevent ALD (Figure 5). Furfural is also commonly found in most alcoholic beverages (Alcázar et al., 2006), which may explain the reason that certain alcoholic beverages consumed in daily life are less damaging than others.

## Conclusion

In conclusion, in this study, we explored the NAD^+^/NADH redox state and mitochondrial function after treatment of mice and cells with ethanol and furfural. An appropriate amount of furfural led to the recovery of the balanced redox state, affecting metabolic pathways and maintaining mitochondrial function. These findings may prompt a neoteric application to the prevention and treatment of ALD.

## Data Availability

The datasets presented in this study can be found in online repositories. The names of the repository/repositories and accession number(s) can be found below: https://ngdc.cncb.ac.cn/gsa-human; HRA002245.

## Abbreviations

ALD, alcohol-associated liver disease; NAD, nicotinamide adenine dinucleotide; DEGs, differentially expressed genes; GO, gene ontology; KEGG, kyoto encyclopedia of genes and genomes; BP, biological process; CC, cellular component; MF, molecular function; IGFBP3, insulin-like growth factor binding protein 3; IGF-1 (R), insulin-like growth factor 1 (receptor); IL-11, interleukin-11; TGF-β1, transforming growth factor-beta 1; PI3K, phosphoinositide 3-kinase; mTOR, mammalian target of rapamycin; Apaf-1, apoptotic peptidase activating factor 1; CytC, cytochrome c; SOD2, superoxide dismutase 2; CAT, catalase; GSH-Px1, glutathione peroxidase 1; 5-HMF, 5-hydroxymethyl-2-furfural.
