# Supplementary material for: Furfural Produces Dose-Dependent Attenuating Effects on Ethanol-Induced Toxicity in the Liver
Source: Front Pharmacol. 2022 Jun 8;13:906933. doi: 10.3389/fphar.2022.906933 (PMC9214037; doi:10.3389/fphar.2022.906933)
Supplement: Supplementary file 2 [file Table1.DOCX]

Supplementary Table 1. Quantitative real-time PCR primers

| Gene | Forward primer | Reverse primer |
| --- | --- | --- |
| IGFBP3 | CGCTACAAAGTTGACTACGAGTC | GTCTTCCATTTCTCTACGGCAGG |
| IGF-1 | CTCTTCAGTTCGTGTGTGGAGAC | CAGCCTCCTTAGATCACAGCTC |
| IGF-1R | ACGCCAATAAGTTCGTCCACAGAGACCT | GAAGACTCCATCCTTGAGGGACTCAG |
| Caspase-9 | ATGATCGAGGACATCCAGCG | GGCCTGTGTCCTCTAAGCAG |
| Apaf-1 | AGTGGTAAAGATTCAGTTAGTGGAA | TGACCCATCCTGGTTCACCT |
| CytC | ATGGTCTCTTTGGGCGGAAG | TCACACTCCTGATAGTTTGCCA |
| IL11 | CGAGCGGACCTACTGTCCTA | GCCCAGTCAAGTGT CAGGTG |
| TGF-$\beta1$ | TGAGGGCTTTCGCCTTAGC | CGGTAGTGAACCCGTTGATGT |
| TGF-$\beta1$R | CTCCAAACCACAGAGTGGGAA | ATCATCGAGAACTTCAGGGGC |
| Bcl-2 | ATCGCCCTGTGGATGACTGAGT | GCCAGGAGAAATCAAACAGAGGC |
| Bax | TCTGACGGCAACTTCAACTG | TGGGTGTCCCAAAGTAGGAG |
| PGC-1$\alpha$ | AGCTGAGTGTTGGCTGGTGCC | CCCTCCACCCCAGGAGGCAG |
| SOD2 | CTGGACAAACCTCAGCCCTAAC | AACCTGAGCCTTGGACACCAAC |
| CAT | GTGCGGAGATTCAACACTGCCA | CGGCAATGTTCTCACACAGACG |
| GSH-Px1 | GTGCTCGGCTTCCCGTGCAAC | CTCGAAGAGCATGAAGTTGGGC |
| GAPDH | GTCTCCTCTGACTTCAACAGCG | ACCACCCTGTTGCTGTAGCCAA |
