# Supplementary material for: Furfural Produces Dose-Dependent Attenuating Effects on Ethanol-Induced Toxicity in the Liver
Source: Front Pharmacol. 2022 Jun 8;13:906933. doi: 10.3389/fphar.2022.906933 (PMC9214037; doi:10.3389/fphar.2022.906933)
Supplement: Supplementary file 4 [file DataSheet1.docx]

Supplementary Material

# Supplementary Figures and Tables

## Supplementary Figures


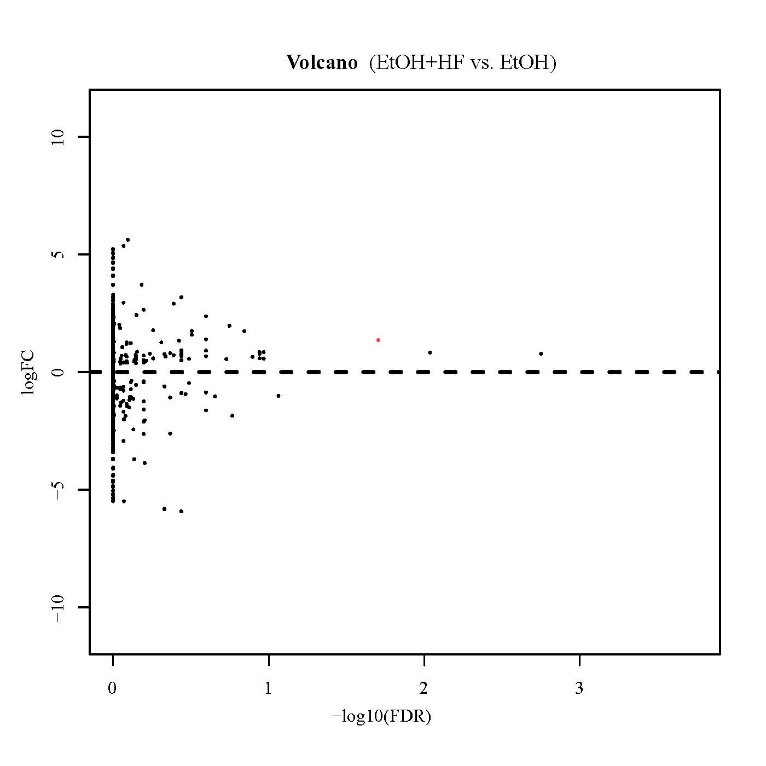


**Supplementary Figure 1.** Volcano plot showing the DEGs between the EtOH+HF group and the EtOH group.


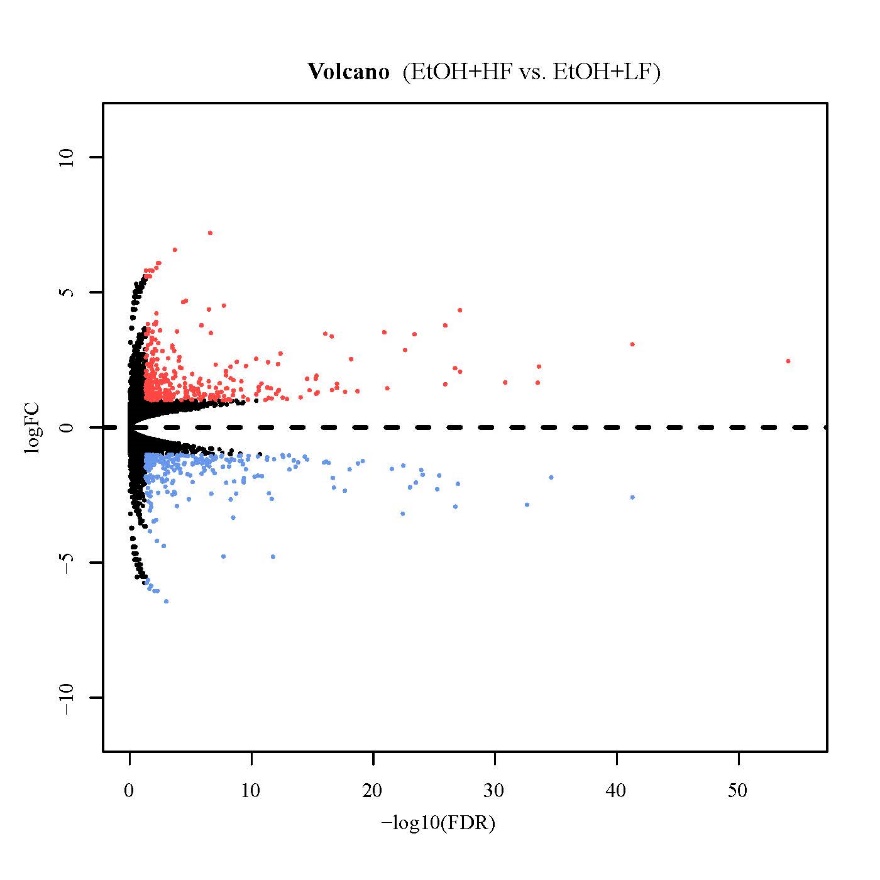


**Supplementary Figure 2.** Volcano plot showing the DEGs between the EtOH+HF group and the EtOH+LF group.


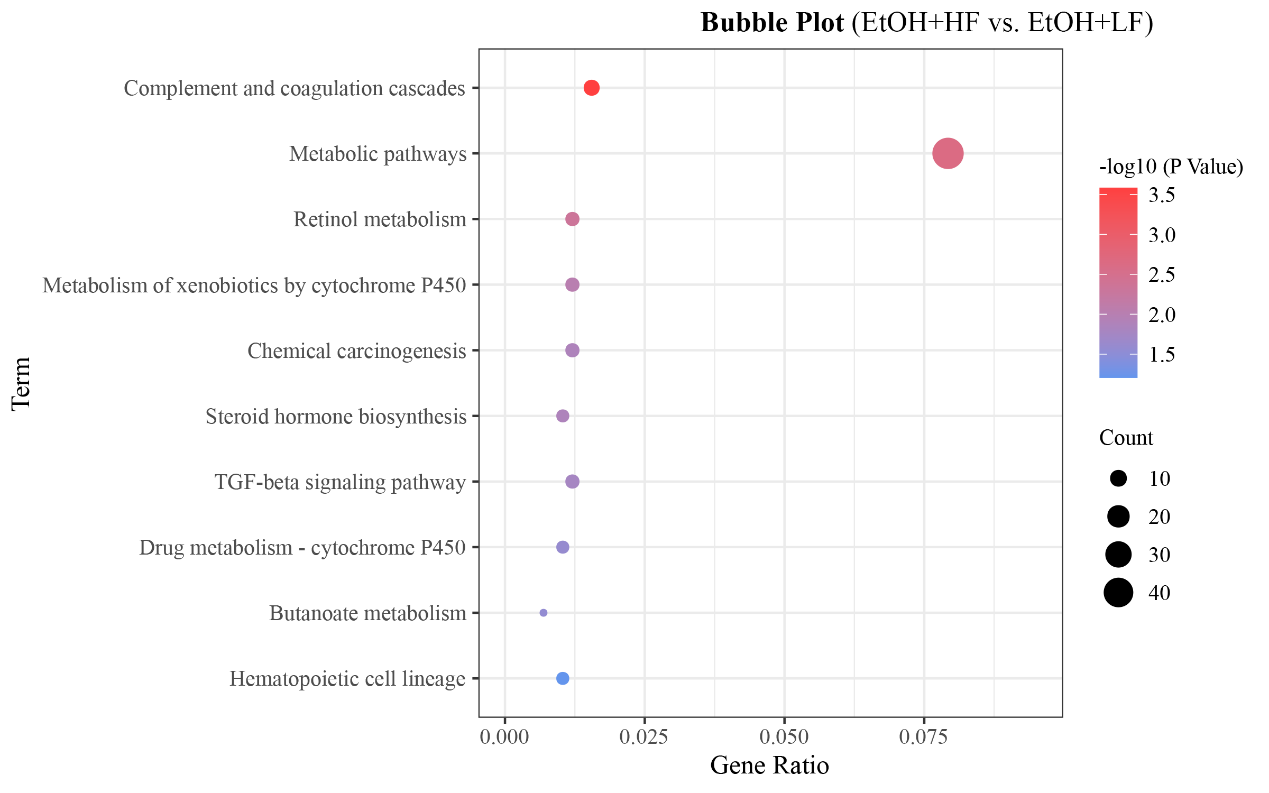


**Supplementary Figure 3.** KEGG pathway enrichment analysis between the EtOH group and the EtOH+HF group


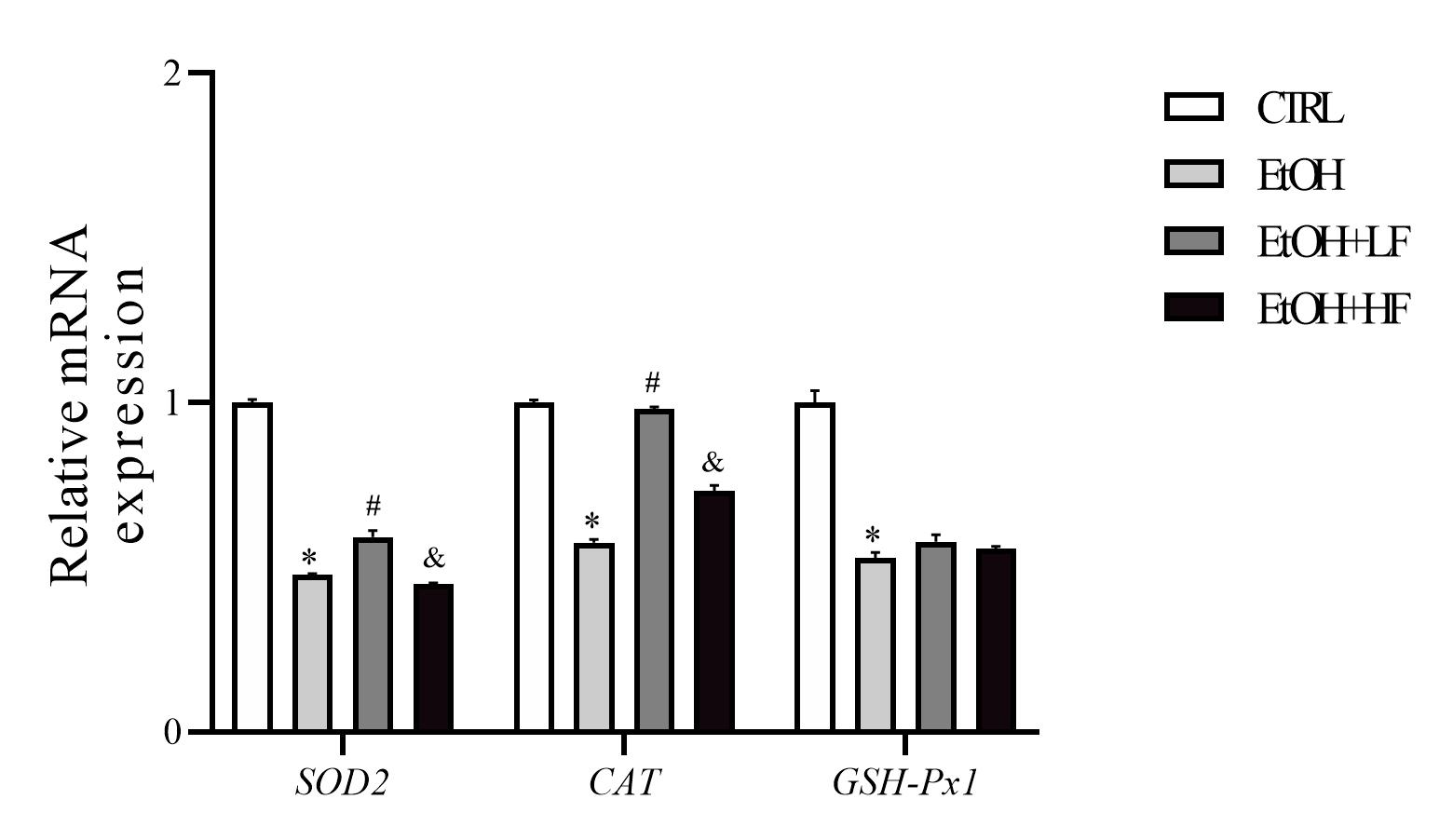


**Supplementary Figure 4.** The mRNA levels of antioxidant enzymes as measured by RT–qPCR
